# CCAAT/enhancer-binding protein α is required for hepatic outgrowth via the p53 pathway in zebrafish

**DOI:** 10.1038/srep15838

**Published:** 2015-10-29

**Authors:** Hao Yuan, Bin Wen, Xiaohui Liu, Ce Gao, Ruimeng Yang, Luxiang Wang, Saijuan Chen, Zhu Chen, Hugues de The, Jun Zhou, Jun Zhu

**Affiliations:** 1 CNRS-LIA124, Sino-French Research Center for Life Sciences and Genomics, State Key Laboratory of Medical Genomics, Rui-Jin Hospital, Shanghai Jiao Tong University School of Medicine, Shanghai, China; 2 Key Laboratory for Molecular Animal Nutrition, Ministry of Education, College of Animal Sciences, Zhejiang University, Hangzhou, China; 3 Université de Paris 7/INSERM/CNRS UMR 944/7212, Equipe Labellisée No. 11 Ligue Nationale Contre le Cancer, Hôpital St. Louis, Paris, France

## Abstract

CCAAT/enhancer-binding protein α (C/ebpα) is a transcription factor that plays important roles in the regulation of hepatogenesis, adipogenesis and hematopoiesis. Disruption of the *C/EBPα* gene in mice leads to disturbed liver architecture and neonatal death due to hypoglycemia. However, the precise stages of liver development affected by C/ebpα loss are poorly studied. Using the zebrafish embryo as a model organism, we show that inactivation of the *cebpa* gene by TALENs results in a small liver phenotype. Further studies reveal that C/ebpα is distinctively required for hepatic outgrowth but not for hepatoblast specification. Lack of C/ebpα leads to enhanced hepatic cell proliferation and subsequent increased cell apoptosis. Additional loss of p53 can largely rescue the hepatic defect in *cebpa* mutants, suggesting that C/ebpα plays a role in liver growth regulation via the p53 pathway. Thus, our findings for the first time demonstrate a stage-specific role for C/ebpα during liver organogenesis.

Hepatogenesis in mammals has been studied extensively in recent years. In mice, liver organogenesis initiates from the ventral foregut endoderm at embryonic day 8.0 (e8.0), followed by the specification of definitive hepatoblasts. At e9.5, the hepatoblasts delaminate from the epithelium and invade the septum transversum mesenchyme (STM) to form the liver bud. Between e9.5 and e15, the liver bud undergoes rapid proliferation. Finally, the hepatoblasts differentiate into mature hepatocytes and biliary duct cells^1^,^2^. In addition, it is worth noting that the fetal liver of mammals is also a major hematopoietic organ^3^.

Over the last decade, the zebrafish (*Danio rerio*) has emerged as a favored model organism in hepatogenesis^4^. In zebrafish, hepatogenesis can be divided into three main stages: specification, budding/differentiation and hepatic outgrowth. Hepatoblasts originate from the anterior endoderm and can be recognized by the expression of *hhex* and *prox1* as early as 22–24 hours post fertilization (hpf). Following this, hepatoblast differentiation occurs within the liver primordium by 50 hpf, and can be clearly distinguished as a prominent bud settling on the left side of the midline over the yolk. At the subsequent hepatic outgrowth stage beginning at 60 hpf, the liver dramatically expands because of rapid cell proliferation. By the end of outgrowth at 5 days post fertilization (dpf), the functional liver consists of a larger left lobe and a smaller right lobe^5^,^6^. While many of the transcription factors and signaling pathways essential for the first two stages have been identified^5^, much less is known about the genes required for hepatic outgrowth.

C/ebpα is the founding member of C/EBP family of basic leucine zipper (bZIP) transcription factors^7^. It exerts important biological functions in a range of cell types^8^. Targeted disruption of the *C/EBPα* gene in mice leads to abundant generation of pseudoglandular structures in the liver parenchyma during perinatal liver development^9^,^10^. The knockout mice die shortly after birth because of hypoglycemia accompanied by hyperammonemia^9^,^10^,^11^. Although C/EBPα is implicated in transcriptional regulation of genes for enzymes of gluconeogenesis and urea synthesis in the liver, the precise stages that are affected by C/EBPα deficiency during liver development have not been fully elucidated.

In the present study, we show that disruption of the *cebpa* gene using TALENs results in a small liver phenotype in zebrafish. Lack of *cebpa* blocks hepatic outgrowth, but does not affect liver specification. C/ebpα regulates hepatic cell proliferation and apoptosis during liver outgrowth. Furthermore, loss of p53 can largely rescue the hepatic defect in *cebpa* mutant embryos, suggesting a novel link between C/ebpα and the p53 pathway in the regulation of liver development.

## Results

### Disruption of zebrafish *cebpa* gene by TALENs

**Figure 1:**
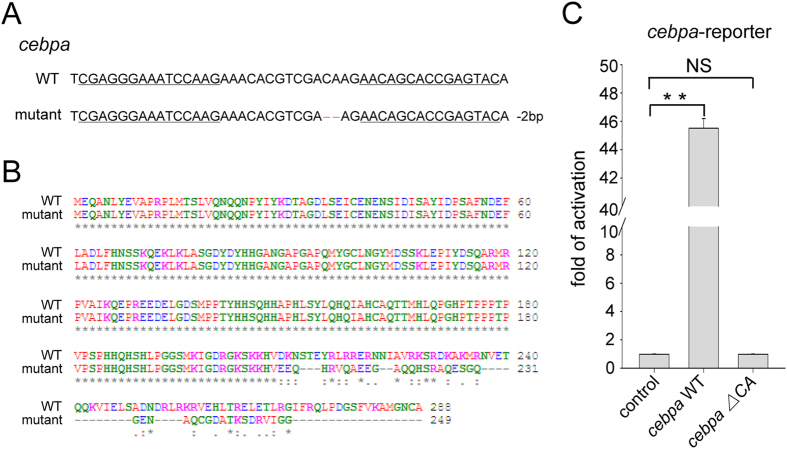
Inactivation of the zebrafish *cebpa* gene based on TALENs. (**A**) Partial nucleotide sequences of the wild type and mutant *cebpa* genes. The TALENs binding sites are underlined. Deletions are indicated by red dashes. (**B**) Amino acid sequence alignment of wild type and mutant C/ebpα proteins. (**C**) Luciferase activity assays were performed in 293T cells using C/ebpα constructs indicated. Renilla was used as an internal control. Data shown are the mean ± SD of three independent experiments, **P < 0.005 by student’s *t*-test. NS, not significant. Note that the mutant C/ebpα (ΔCA), with two nucleotide deletions, loses its transcriptional activity.

We have previously generated a *cebpa* mutant (*cebpa*^*rj31/+*^) using TALENs technology^12^. In this mutant, two nucleotides were deleted at the *cebpa* locus (*cebpa ∆CA*) (Fig. 1A), which led to frame shift at amino acid residue 209. This mutation created a premature stop codon, resulting in the synthesis of a truncated C/ebpα lacking the bZIP domain at the C-terminus (Fig. 1B). Luciferase assay showed that the mutant C/ebpα completely lost its transcriptional activity compared with the wild type (Fig. 1C), confirming inactivation of the mutant C/ebpα. The *cebpa* mutant zebrafish generally died around 2 to 3 weeks post fertilization.

### The *cebpa* mutant exhibits a small liver phenotype

**Figure 2:**
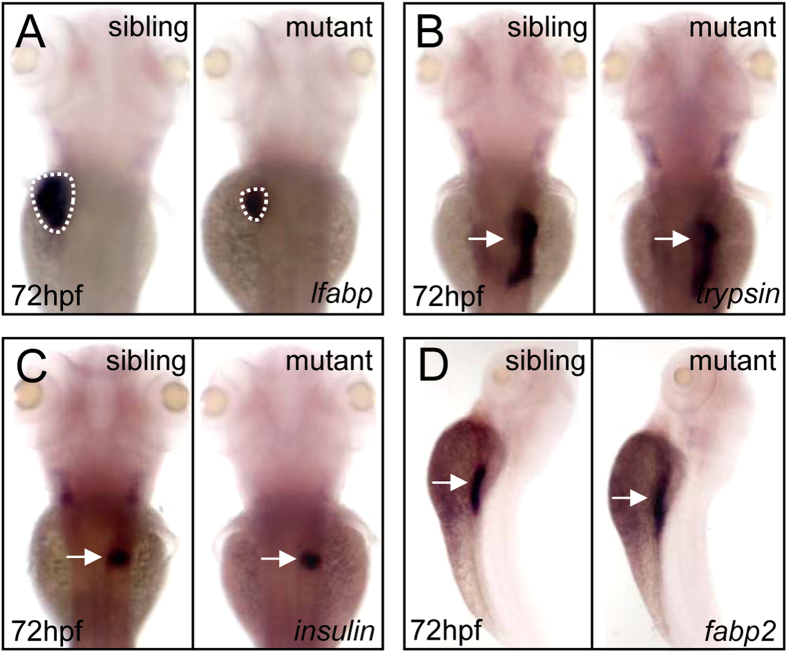
Disruption of *cebpa* gene reduces liver size. (**A**–**D**) WISH assay of *lfabp* (**A**), *trypsin* (**B**), *insulin* (**C**) and *fabp2* (**D**) at 72 hpf, respectively. Dashed lines circle the boundary of the liver. (**A**–**C**), dorsal views, anterior to the top. (**D**) lateral view, dorsal to the right.

To study the function of C/ebpα in embryonic liver development, we first analyzed the expression pattern of *cebpa* by whole mount *in situ* hybridization. The results showed that *cebpa* expression was enriched in the developing liver (supplemental Fig. S1), consistent with the previous observation^13^. We then examined the expression of *liver fatty acid binding protein* (*lfabp*), a liver-specific marker^14^. The data revealed that the liver size of the *cebpa* mutant was strikingly reduced, compared to that of sibling controls at 72 hpf and 5 dpf, respectively (Fig. 2A and supplemental Fig. S2). In contrast, the development of other endoderm-derived tissues such as exocrine pancreas, endocrine pancreas and intestine was not obviously affected as determined by assessing the expression of *trypsin*, *insulin* and *fatty acid binding protein 2* (*fabp2*), respectively (Fig. 2B–D). Together, these results suggest that *cebpa* is essential for liver development in zebrafish.

### C/ebpα is required for hepatic outgrowth but not for hepatoblast specification

**Figure 3:**
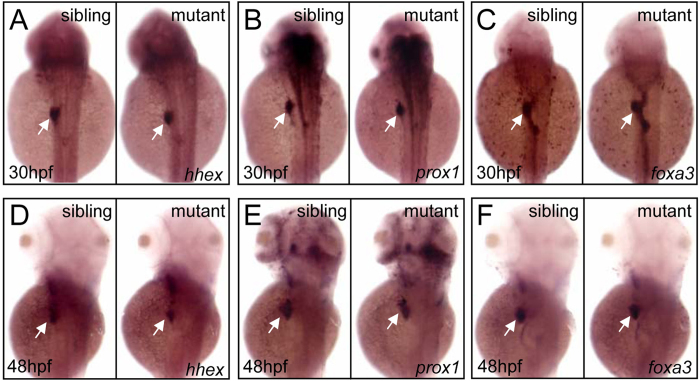
*cebpa* is dispensable for liver specification or budding. (**A**–**F**) WISH assay of *hhex* (**A** and **D**), *prox1* (**B** and **E**) and *foxa3* (**C** and **F**) at 30 and 48 hpf, respectively. (**A**–**F**), dorsal views, anterior to the top. White arrows indicate liver primordium.

The failure of liver development in the *cebpa* mutant could be attributable to defects in hepatoblast specification from endodermal cells or budding prior to hepatic outgrowth. To test which stage had been affected, we examined the expression of *hhex* and *prox1*, representing the earliest markers for hepatoblasts^15^,^16^. Data showed that these genes displayed similar expression patterns in the liver primordia of both sibling and mutant embryos at 30 hpf (completion of specification) (Fig. 3A,B) and 48 hpf (completion of budding) (Fig. 3D–E). Moreover, the expression of *foxa3*, a pan-endodermal marker, was also unaffected in the mutant embryos (Fig. 3C,F). Therefore, these results, together with that of *lfabp*, indicate that C/ebpα is not required for liver specification but for liver expansion growth during hepatogenesis.

### *cebpa* deficiency results in enhanced hepatic cell proliferation and subsequent increased apoptosis

**Figure 4:**
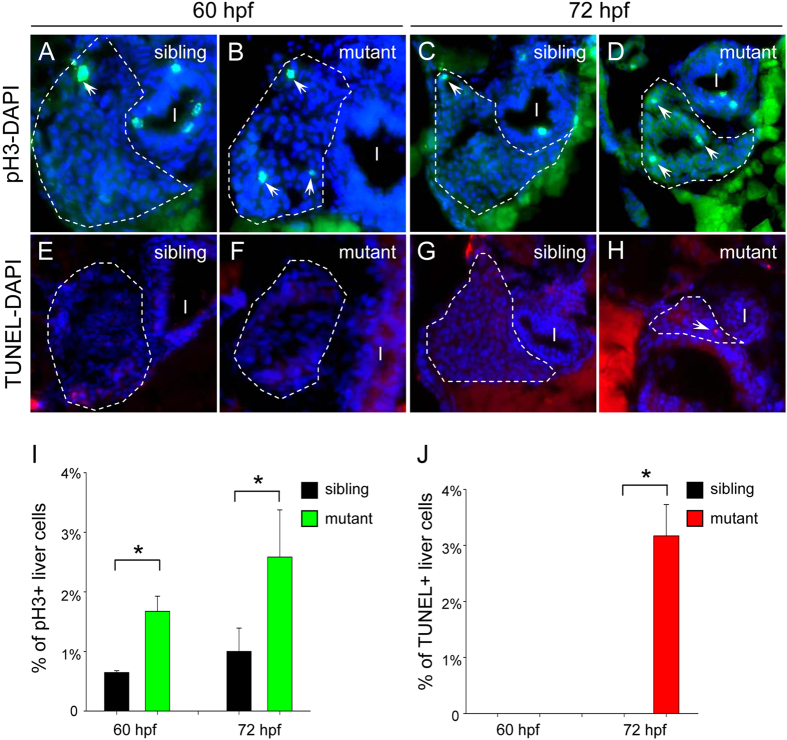
Loss of *cebpa* leads to enhanced hepatic cell proliferation and subsequent increased apoptosis. (**A**–**H**) Hepatic cell proliferation and apoptosis were determined by pH3 staining and TUNEL assay in 60 and 72 hpf embryos, respectively. The sections were counterstained with DAPI to label the nucleus. Dashed lines circle the boundary of the liver. White arrows indicate pH3 or TUNEL positive cells, respectively. In each case or at each time-point, more than 5 sections from at least three sibling control or *cebpa* mutant fish were examined. (**I**) intestine. (**I**,**J**) Quantification of hepatic cell proliferation and apoptosis, respectively. Data shown are the mean ± SD, n ≥ 3, *P < 0.05 by student’s *t*-test.

The small liver phenotype in the *cebpa* mutant could potentially result from abnormal cell proliferation and/or apoptotic cell death. We next examined the terminal fate of the *cebpa*-deficient hepatic cells using immunostaining. Firstly, using an antibody against phospho-histone H3 (pH3), a marker of cell proliferation, we found that the pH3-positive hepatic cells exhibited a 2.5-fold increase in the *cebpa* mutant sectioned embryos at 60 and 72 hpf, respectively (Fig. 4A–D,I). Next, we examined apoptotic events using the TUNEL assay. The mutant hepatic cells underwent significant activation of cell apoptosis at 72 hpf, whereas almost no apoptotic cells were detected in the developing liver of sibling controls or at earlier developmental stage (Fig. 4E–H,J). Collectively, these results suggest that loss of C/ebpα results in enhanced hepatic cell proliferation and subsequent increased cell apoptosis which may account for the small liver phenotype.

### p53 pathway activation in the *cebpa* mutant

**Figure 5:**
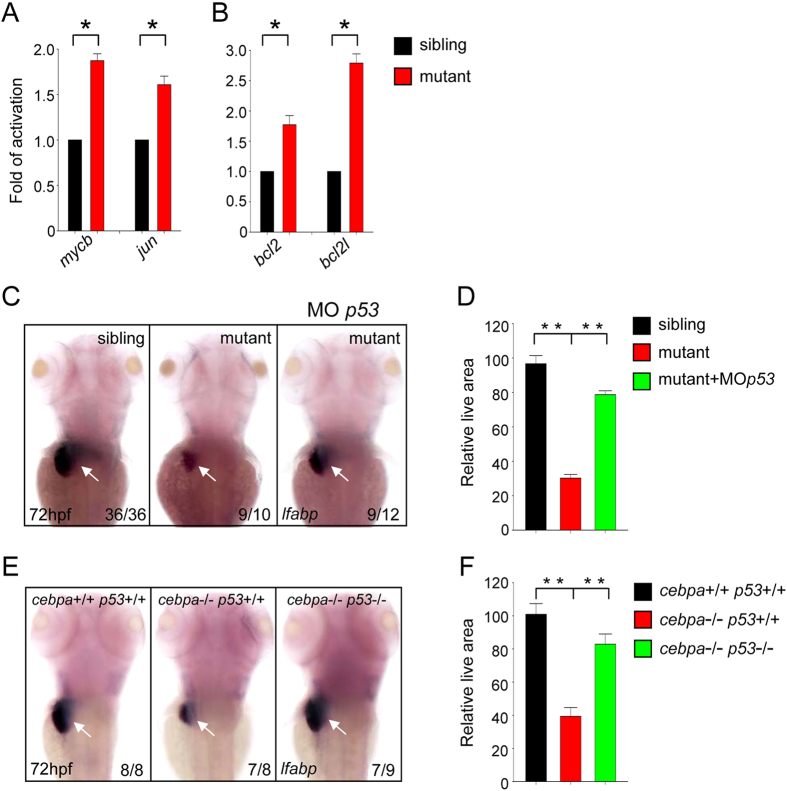
The *p53* signaling pathway is activated in *cebpa*-deficient developing liver. (**A**–**B**) Quantitative PCR analysis of the expression of cell proliferation and apoptosis-related genes in 72 hpf embryos. Data shown are the mean ± SD, n ≥ 3, *P < 0.05 by student’s *t*-test. (**C**,**E**) WISH assay of *lfabp* at 72 hpf. Loss of *p53* could rescue the hepatic defect in *cebpa* mutant embryos. Dorsal views with anterior to the top. (**D**,**F**) The relative liver area measured in (**C**,**E**) respectively. The result shown is fold difference compared with the level (set to 100) detected in control embryos (mean ± SD, n ≥ 3, **P < 0.005 by student’s *t*-test).

To study the molecular mechanisms that may underlie the small liver phenotype in the *cebpa* mutant, we examined the expression levels of genes which are involved in cell proliferation and apoptosis. It was previously shown that the expression of *c-myc* and *c-jun* were induced in the liver of *C/EBP*α knockout mice^10^. In agreement with this, we also detected elevated expression of *mycb* and *jun* in the *cebpa* mutant zebrafish embryos (Fig. 5A), underscoring the evolutionary conserved role of these genes in the regulation of normal liver development. Importantly, we also observed that the expression of *bcl2* and *bcl2l* were significantly increased in these mutant embryos (Fig. 5B), supporting the notion that an increased portion of the *cebpa-*deficient hepatic cells were in an apoptotic state. It is well known that the p53 signaling pathway plays a key role in controlling cell proliferation and apoptosis^17^. The aberrant cell proliferation and apoptosis observed in the *cebpa* mutant suggests that p53 pathway is likely activated. In order to test this, we knocked down p53 using morpholino (MO) in the *cebpa* mutant embryos. As expected, the hepatic defect of the *cebpa* mutant embryos could be efficiently rescued by p53 knockdown (Fig. 5C,D). To further confirm the role of p53 in this process, we next generated *cebpa* and *p53* double mutant zebrafish. The data showed that additional loss of p53 could restore normal liver development in *cebpa*-deficient embryos (Fig. 5 E,F). Moreover, the defects of cell proliferation and apoptosis could be also largely recovered in the *cebpa* and *p53* double mutant (supplemental Fig. S3). Thus, these results demonstrate that the p53 pathway is indeed involved in C/ebpα-dependent hepatic outgrowth.

## Discussion

Targeted disruption of the *C/EBP*α gene in mice leads to disturbed liver architecture and results in neonatal death due to hypoglycemia^9^,^10^. However, the exact stages affected by C/EBPα deficiency during liver development have not been analyzed in detail. Furthermore, the fetal liver of mammals is also a hematopoietic organ, and hepatogenesis and hematopoiesis are intertwined. Therefore, dysfunction of one process may prevent the proper development of the other. C/EBPα not only plays important roles in hepatogenesis, but also is required for hematopoiesis^18^, which obfuscates its role in these two processes. To assess the independent function of C/EBPα in liver development, we used the popular model organism, zebrafish, due to the fact that embryonic hematopoiesis does not take place in the liver. Here, we found that the expression of *cebpa* was enriched in the developing liver of zebrafish. Inactivation of the *cebpa* gene by TALENs led to a small liver phenotype. Detailed analysis revealed that C/ebpα was required for hepatic outgrowth but not for hepatoblast specification during liver development.

An increasing number of reports have implicated C/EBPα as a suppressor of cell proliferation^8^. In support of this idea, we found that loss-of-function of C/ebpα induced hepatic proliferation in the developing liver of zebrafish. Interestingly, we also detected increased hepatic cell apoptosis in the *cebpa* mutant embryos at later developmental stage. These ambivalent results suggest that the *cebpa*-deficient hepatic cells seemed to be in an inappropriate proliferative state and then underwent apoptosis, eventually resulting in the small liver phenotype. It is not a rare phenomenon in the liver development, since disruption of *Apc* (*adenomatous polyposis coli*) in the liver of mice also leads to increased hepatocyte proliferation and apoptosis, which may be caused by elevated DNA damage, accumulation of p53 and increased levels of anaphase bridges^19^. Additionally, in the partial hepatectomy (PH)-induced mouse liver regeneration study, *Nur77* knock-out livers exhibited enhanced hepatocyte proliferation coincided with hepatocyte apoptosis^20^. Further studies are required to investigate the switch between cell proliferation and apoptosis regulated by C/ebpα, such as the role of C/ebpα in controlling chromosome segregation and genomic stability.

The p53 pathway is composed of a network of genes responding to a variety of intrinsic and extrinsic stress signals. Activation of the p53 protein induces cell cycle arrest, cellular senescence or apoptosis^17^,^21^. p53, as a well-known tumor suppressor, also plays a critical role during organogenesis, including hepatogenesis. In zebrafish *def*^*hi429*^ (digestive-organ expansion factor) mutant, the expression of ∆*113p53*, a newly identified isoform of p53, was selectively up-regulated within the mutant digestive organs, and then triggered the arrest of cell proliferation, resulting in compromised organ growth. Furthermore, knock-down of p53 and Δ 113p53 levels could rescue the developmental defects of the mutants^22^,^23^. Moreover, the Def-p53 pathway was also involved in scar formation at the amputation site after PH in zebrafish^24^. Here, we showed that the p53 pathway was activated in the *cebpa*-deficient embryos, which may be triggered by the aberrant cell proliferation, and additional loss of p53 could largely rescue the hepatic defects in the *cebpa* mutants. However, *p53* might not be a direct target gene of C/ebpα in the liver organogenesis, since the transcriptional level of *p53* has no obvious changes in the *cebpa* mutants compared with sibling controls (supplemental Fig. S4). It will be of interest in future studies to determine how C/ebpα regulates p53 activities in the developing liver.

Taken together, we hereby provide novel evidence that C/ebpα is specifically required for hepatic outgrowth via the p53 pathway, and accordingly have expanded our understanding of liver development. Moreover, these new findings may help to identify new targets for therapeutic manipulation in the treatment of liver failure and liver cancer.

## Methods

### Zebrafish

Zebrafish maintenance and staging were performed as described previously^25^. The zebrafish facility and study were approved by the Institutional Review Board of the Institute of Health Sciences, Shanghai Institutes of Biological Sciences, Chinese Academy of Sciences (Shanghai, China) and the methods were carried out in accordance with the approved guidelines. The *cebpa*^*rj31/+*^ and *p53*^*zdf1/zdf1*^ mutants were used in this study^12^,^26^.

### Generation of constructs

The zebrafish *cebpa∆CA* was generated by genomic PCR with the following primers: Forward 5’ CCGGAATTCATGGAGCAAGCAAACCTCTACGAGG 3’, Reverse 5’ CCGCTCGAGTTAAGCGCAGTTGCCCATGGCTTTGAC 3’, then cloned into the pCS2+ vector.

### Luciferase reporter assay

293T cells were transfected with the indicated plasmids using Effectene Transfection Reagent (QIAGEN). Tetramer of the *CEBP* site of human *GCSFR* was inserted into the promoterless luciferase vector pTK81-luc and used as a reporter plasmid^27^. Cells were harvested 36 hours after transfection and luciferase activities were analyzed using the Dual Luciferase Reporter Assay Kit (Promega), according to the manufacturer’s protocols. Luciferase activity was normalized to Renilla activity.

### Morpholino

The morpholino oligonucleotide (MO) of p53 (TCTTGGCTGTCGTTTTGCGCCATTG) was used as previously described^28^.

### Whole-mount mRNA *in situ* hybridization

Digoxigenin-labeled antisense RNA probes were transcribed from linearized constructs using T3, T7 or Sp6 polymerase (Roche). Whole-mount mRNA *in situ* hybridization was performed as described previously^18^. The probes were detected using alkaline phosphatase (AP)-coupled anti-digoxigenin Fab fragment antibody (Roche) with BCIP/NBT staining (Vector Laboratories). The probes used in this study included: *cebpa*, *lfabp*, *trypsin*, *insulin*, *fabp2*, *hhex*, *prox1* and *foxa3*.

### Phospho-histone H3 (pH3) immunostaining and TUNEL assay

Embryos were fixed in 4% paraformaldehyde at 4 °C overnight. For sectioning, the embryos were embedded in OCT compound (SAKURA) and cryosectioned into 14μm slices. After blocking with 10% FBS for 1 hr at room temperature, the sections were incubated with rabbit anti-pH3 antibody (1:100 dilution, Santa Cruz) at 4 °C overnight. Secondary antibody of Alexa Fluor 488 conjugated anti-rabbit IgG (Invitrogen) was then incubated for 1 hr at room temperature. The sections were counterstained with DAPI (Vector Labs) to label cell nuclei.

Terminal transferase UTP nick end labeling (TUNEL) was carried out on cryosections using the *In Situ* Cell Death Detection Kit, TMR red (Roche) according to the manufacturer’s recommendations.

### Quantitative PCR

Total RNA was extracted from the head region containing liver dissected from embryos at 72 hpf, and the trunks of the embryos were used for genotyping. Quantitative PCR was performed using a LightCycler 1.5 (Roche) following the manufacturer’s protocol. Primers are listed in supplemental Table S1.

## Additional Information

**How to cite this article**: Yuan, H. *et al.* CCAAT/enhancer-binding protein a is required for hepatic outgrowth via the p53 pathway in zebrafish. *Sci. Rep.* **5**, 15838; doi: 10.1038/srep15838 (2015).

## Supplementary Material

Supplementary Information
