# Supplementary material for: CCAAT/enhancer-binding protein α is required for hepatic outgrowth via the p53 pathway in zebrafish
Source: Sci Rep. 2015 Oct 29;5:15838. doi: 10.1038/srep15838 (PMC4649991; doi:10.1038/srep15838)
Supplement: Supplementary Information [file srep15838-s1.pdf]

CCAAT/enhancer-binding protein α is required for hepatic outgrowth via the p53 pathway in zebrafish

Hao Yuan[1], Bin Wen[1], Xiaohui Liu[1], Ce Gao[2], Ruimeng, Yang[1], Luxiang Wang[1], Saijuan Chen[1], Zhu Chen[1], Hugues de The[1, 3], Jun Zhou[1, *] and Jun Zhu[1, 3, *]

Supplemental figure legends

Fig. S1. *cebpa* is expressed in the developing liver. (A-C) WISH assay of *cebpa* from 24 to 48 hpf. White arrows indicate the liver-forming region. A-C, lateral views, dorsal to the right. A'-C', dorsal views, anterior to the top.

Fig. S2. *cebpa* mutant embryos display a small liver phenotype. (A-B) WISH assay of *lfabp* at 5 dpf. Dashed lines circle the boundary of the liver. Lateral views, dorsal to the top.

Fig. S3. Additional loss of p53 could rescue the defects of cell proliferation and apoptosis observed in *cebpa*-deficient liver. (A-F) Hepatic cell proliferation and apoptosis were determined by pH3 staining and TUNEL assay in 72 hpf embryos, respectively. The sections were counterstained with DAPI to label the nucleus. Dashed lines circle the boundary of the liver. White arrows indicate pH3 or TUNEL positive cells, respectively. In each case, more than 5 sections from at least three sibling control or *cebpa-p53* double mutant fish were examined. I, intestine. (G-H) Quantification of hepatic cell proliferation and apoptosis, respectively. Data shown are the mean ± SD, n≥3, *$P<0.05$ by student's *t-test*. NS, not significant.

Fig. S4. The transcriptional level of *p53* was unaffected in *cebpa* mutant. (A) Quantitative PCR analysis of the expression of *p53* in 72 hpf embryos. Data shown are the mean ± SD of three independent experiments. NS, not significant.

Figure S1

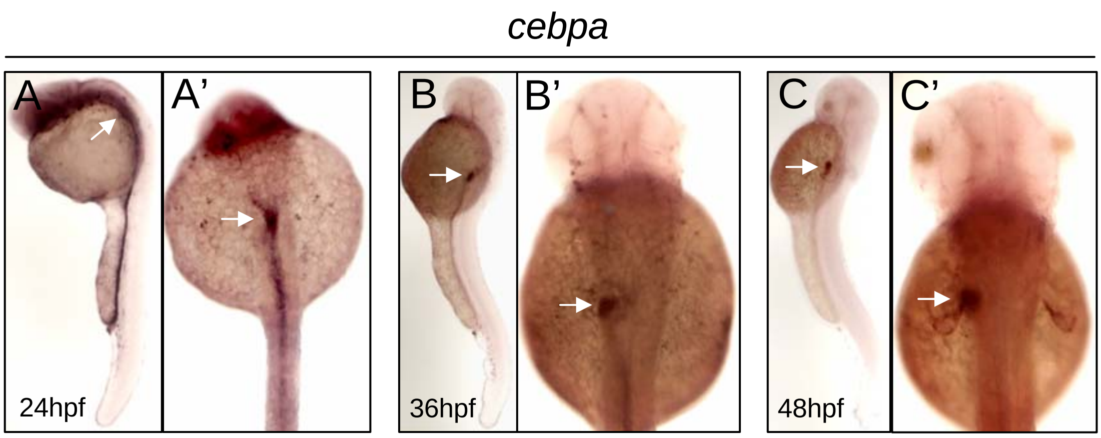

Figure S2

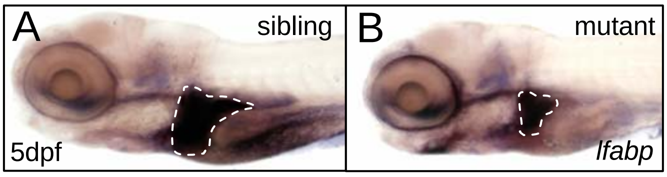

Figure S3

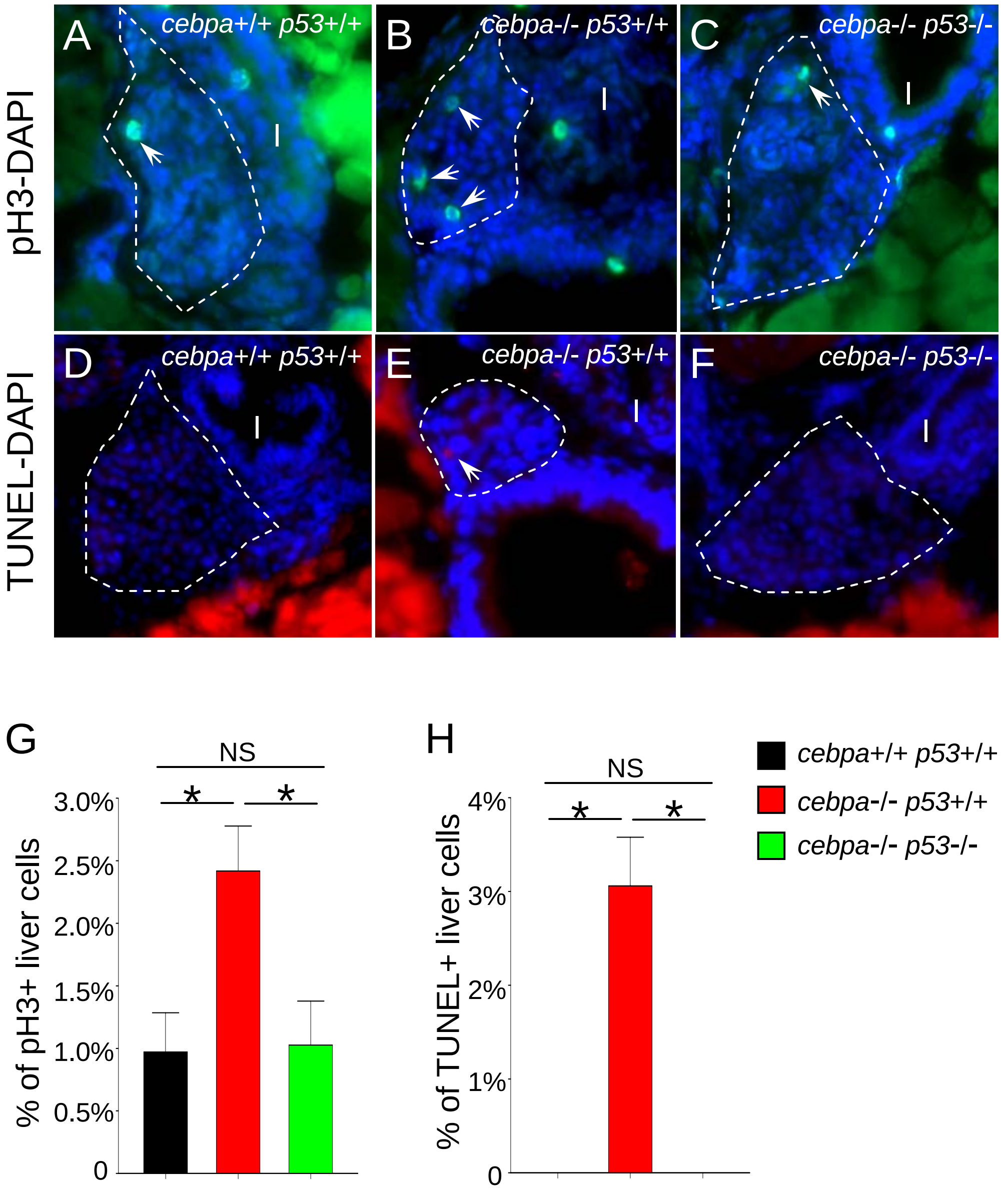
A
cebpa+/+ p53+/+
B
cebpa-/- p53+/+
C
cebpa-/- p53-/-
pH3-DAPI
D
cebpa+/+ p53+/+
E
cebpa-/- p53+/+
F
cebpa-/- p53-/-
TUNEL-DAPI
I
G
NS
*
3.0%
2.5%
2.0%
1.5%
1.0%
0.5%
0
% of pH3+ liver cells
H
NS
4%
3%
2%
1%
0
% of TUNEL+ liver cells
cebpa+/+ p53+/+
cebpa-/- p53+/+
cebpa-/- p53-/-

Figure S4

A

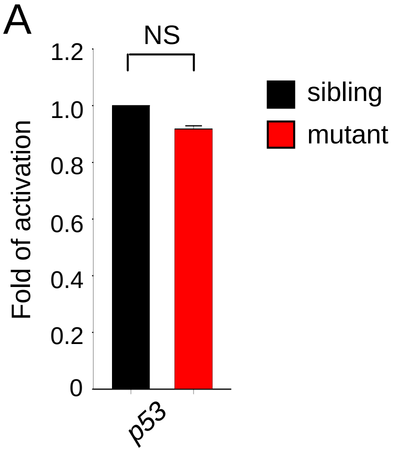

Table S1. Primer sequences of quantitative PCR.

Gene	primer sequences
jun	AGGAGAGCCGAAGGACATTT
	CAGACATCAAGCCCCTGAA
mycb	AGCAGTAGTGACAGCGAATCC
	CCGTGACCACGTCAATTTCT
bcl2	TGAGGCTCTACCGGGTGTTA
	TCGCGTTGGTAAATCCTTTC
bcl2l	GGCTTGTTTGCTTGGTTGAC
	TGGTGCAATGGCTCATACC
gapdh	TTGAGAAACCTGCCAAGTATGA
	CCCATTGAAGTCAGTGGACA
